# Two-year safety and efficacy of Indigenous Abluminus Sirolimus Eluting Stent. Does it differ amongst diabetics? – Data from en-ABLe- REGISTRY

**DOI:** 10.34172/jcvtr.2021.31

**Published:** 2021-05-19

**Authors:** Kamal Sharma, Sameer Dani, Devang Desai, Prathap Kumar, Nirav Bhalani, Apurva Vasavada, Rutvik Trivedi

**Affiliations:** ^1^Department of Cardiology, UNMICRC, BJ Medical College, Ahmedabad (Gujarat), India; ^2^Apollo Hospitals Ahmedabad and Limsar, Ahmedabad (Gujarat), India; ^3^Unicare Hospital, Mahavir Hospital, Surat (Gujarat) India; ^4^ESIC Hospital Kollam and Meditrina Hospital, Kerala, India; ^5^Rhythm Hopsital and Sunshine Global, Vadodara (Gujarat), India; ^6^Tristar Hopsital and Care Hopsitals, Surat(Gujarat), India; ^7^Zydus Hospital, Anand (Gujarat), India

**Keywords:** Percutaneous Coronary, Interventions, Sirolimus Eluting Stent, Diabetes Mellitus, Abluminus DES+

## Abstract

***Introduction:*** To evaluate the efficacy/safety profile of the Abluminus DES+ over 2-years follow-up in the "real-world" scenario in diabetics as compared to non-diabetics.

***Methods:*** In prospective, all-comers, open-label registry conducted at 31 sites, patients were analyzed for 1 & 2-year outcomes with the primary endpoint defined as 3P-MACE of CV death, target vessel related myocardial infarction (TV-MI), ischemia-driven target lesion revascularization (TLR)/target vessel revascularization (TVR) apart from Stent thrombosis (ST).

***Results:*** Of 2500 patients of PCI with 3286 Abluminus-DES+, 1641 (65.64%) were non-diabetics while859 (34.36%) were diabetics. The 3-P MACE for the cohort at 1 & 2 years were 2.9%, and 3.16%; TLR/TVR - 1.4% at both the intervals for 2493 patients at 2 yrs. follow-up. TV-MI & ST were 0.36% and0.56% at 1st and 2nd year respectively. The 3P-MACE was lower in non-diabetics at 1 & 2 years (2.3%vs 4.2%; 2.4% vs 4.7% respectively). For components of MACE, CV mortality (0.9 vs 1.9% at 1 yr ; 1.0vs 2.1% at 2 years) was significant (*P* < 0.05) while TLR (1.1 vs 1.9% at 1 yr. & 1.1 vs 2.1% at 2 yrs.) and TV-MI (0.9 vs 1.9% at 1 yr. & 1 vs 2.1% at 2 years) were similar for diabetics and non-diabetics so was ST (*P* > 0.05).

***Conclusion:*** Abluminus-DES+ showed excellent 2-year safety and efficacy with low 3-P MACE which was higher in diabetics driven by higher CV death but similar TLR, TV-MI and ST.

## Introduction

The new generation of drug eluting stents (DES) as compared to the older generation of DES have shown better efficacy/safety profile in the treatment of simple as well as complex coronary disease in percutaneous coronary angioplasty (PCI).^1,2^ Nevertheless, the results still have a “residual risk” for certain subsets of high-risk patients, especially amongst the diabetics. The diabetics continue to have worse outcomes following PCI compared with patients without diabetes.^3,4^ This unmet clinical need spurred the introduction of new iterations of DES, theoretically designed to evaluate if they could overcome this “diabetic pitfall”.

A novel Abluminus DES+ - a biodegradable DES with a L605 cobalt-chromium alloy and covered with a biodegradable polymer film, is mounted on a balloon that is specifically designed to deliver a uniform dose of Sirolimus anti-proliferative drug abluminally to the target lesion to reduce restenosis especially in the diabetic patients.

We intend to present the results of the en-ABL-e registry featuring the Abluminus DES+ (Envision Scientific, Surat, India) implanted in the “real-world” scenario. With its unique features it may provide diabetic patients with better short as well as intermediate timeline outcomes as compared to the current generation of DES. Current study is designed to evaluate the efficacy/safety profile of the Abluminus DES+ in the “all-comers” population with minimal exclusion criteria and with a specific focus on the outcomes amongst the diabetic patients as compared to the non-diabetics.

## Materials and Methods

The Envision en-ABL-e registry is a prospective, all comers, multicenter registry that enrolled 2500 patients treated with 3286 Abluminus DES in PCI across 31 centers in India from June 2012 to December 2018. Institutional Ethics committee approvals were obtained from the concerned centers in advance.

### Inclusion criteria

Patients more than 18 years of age; who underwent PCI with ABLUMINUS® DES+ sirolimus eluting stent system; for presence of one or more coronary artery lesion > 70% diameter stenosis in a native coronary artery or a saphenous vein graft ranging from 2.25 to 4.00 mm in diameter that could be covered with one or multiple stents; without any restriction on the number of treated lesions, vessels and lesion length were included in the study.

### Exclusion criteria

Patients who were unable to provide consent; patients needing additional stent not of the study device type (other than Abluminus DES+); patients needing other coronary vascular treatments apart from the ABLUMINUS® DES+ sirolimus eluting stent system viz. drug-eluting balloon or other overlapping DES which could affect the device performance were excluded.

### Procedural technique

#### Pre-procedure

All patients were preloaded with dual antiplatelets, which were at least Aspirin 75mg and clopidogrel bisulphate 300-600 mg. prior to the procedure in drug naïve patients. Patient already on clopidogrel therapy for more than 7 days were not required to be preloaded.

#### During procedure

At least 5000 IU or 70-100 IU/Kg unfractioned heparin was administered to maintain an ACT > 250 seconds during the procedure. DAPT Therapy for a minimum of 12 months was continued as per the standard clinical practice. The use of GP IIB/ IIIA inhibitors was left to the discretion of the operator.

### Follow up

Patients were followed up post-discharge after the index procedure up to 24 months on regular basis. This included telephonic contacts in case the physical consultation was missed; to obtain information regarding medical history, cardiovascular drug usage, any hospitalizations or/and adverse events at 1 month, 3 months, 9 months, 12 months, 24 months and then yearly post procedure. Apart from the clinical follow ups, clinically indicated angiographic follow-up was also encouraged which was left to the discretion of the treating cardiologist.

### Study endpoints

The primary endpoint was defined as a composite of device-oriented 3-point major adverse cardiac events (3P-MACE) of cardiac death, target vessel-related myocardial infarction (MI), and ischemia-driven target lesion revascularization (TLR)/target vessel revascularization (TVR) at 1 and 2 years. Myocardial infarction was always considered target vessel-related, unless angiographically proved that it was not related to the vessel(s) treated with Abluminus DES+. The Stent Thrombosis (ST) rate was calculated at all time points. All the endpoint definitions followed the criteria of the Academic Research Consortium (ARC) for each of the components of 3P-MACE and stent thrombosis.^5^ Periprocedural MI was defined according to the Fourth Universal Definition of MI.^6^

### Statistical analysis

The demographics, risk factors, clinical profile and 3P-MACE at various follow up intervals were captured for the whole cohort and the same was compared between the diabetics and non-diabetics; where quantitative data are being expressed as mean ± SD and qualitative data as percentage. Categorical variables were compared using Pearson’s chi-square test or Fisher’s exact test, whereas difference between continuous variables were assessed using t-test or Mann-Whitney test, as appropriate. A two-sided *P* value of less than 0.05 was considered to indicate statistical significance. All statistical analyses were performed with the use of SPSS vs Version 22.0 (Chicago, IL, USA).

## Results

The baseline characteristics of the diabetic patients are compiled and compared with the non-diabetic patients in Table 1. Demographics, risk factors and clinical factors have been assessed between both the groups. Apart from the older age group in the diabetic cohort (59.4 ± 10.85 vs 56.9 ± 10.85; *P* < 0.001), there was significantly greater prevalence of risk factors such as hypertension (59.6 vs 33.15%), dyslipidemia (8.73 vs 4.26%) renal diseases (3.37 vs 1.84%) with lower baseline left ventricular ejection fraction (LVEF) (39.12 ± 12.22% vs 47.52 ± 8.86%; *P* = 0.0001). History of previous cardiac revascularization (PCI/CABG) was also found to be higher amongst the diabetics as compared to the non-diabetics. The diabetics had smaller stent diameter with small vessels being defined as diameter less than or equal to 2.5 mm and longer stents implantation (defined as > 20 mm) (*P* < 0.0001) as compared to the non-diabetics.

**Table 1:** Baseline characteristics of the cohort amongst Diabetic and non-diabetic patients

**Variables**		**Diabetic (859) Mean ± SD/ N(%)**	**Non-Diabetic (1641) Mean ± SD/ N(%)**	***P*** ** value**
Age (years)		59.4 ± 10.85	56.9 ± 10.85	<0.0001
Male		653 (76.02)	1320 (80)	0.01
Female		206 (24)	321 (20)	
Hypertension		512 (59.60)	544 (33.15)	<0.0001
Smoking/Tobacco		59 (6.86)	116 (7.06)	0.85
Dyslipidemia		75 (8.73)	70 (4.26)	<0.0001
Renal Diseases		29 (3.37)	22 (1.34)	0.0006
Prior CABG		24 (2.79)	26 (1.58)	0.04
Prior PCI		70 (8.14)	78 (4.75)	0.0006
Baseline LVEF(%)		39.12 + 12.22 %	47.52 + 8.86%	0.0001
CHF		3 (0.34)	6 (0.36)	0.95
IHD		137 (15.94)	235 (14.32)	0.28
Prior MI		110 (12.80)	186 (11.33)	0.29
AMI		294 (34.22)	705 (42.96)	<0.0001
ACS		531 (61.81)	1140 (69.46)	0.0001
STEMI		318 (37.01)	753 (45.88)	<0.0001
NSTEMI		35 (4.07)	69 (4.20)	0.87
Unstable Angina		178 (20.72)	318 (19.37)	0.42
Stable Angina		66 (7.68)	89 (5.42)	0.026
Number of Vessels diseased	1	697 (81.14)	1384 (84.33)	0.042
	2	146 (16.99)	224 (13.65)	
	3	16 (1.86)	32 (1.95)	
	4	0	1 (0.06)	
Mean lesion Length (mm)		38.65 ± 11.84	37.8 ± 11.77	0.087
Mean diameter Stenosis (%)		90.43 ± 17.65	91.38 ± 12.09	0.114
Mean Stent Length (mm)		26.66 ± 8.77	26.97 ± 8.45	0.40
Balloon Pre dilation		608 (70.71)	1137 (69.28)	0.44
Total No of Devices	1	608 (70.71)	1257 (76.59)	0.002
	2	199 (23.16)	311 (18.95)	
	3	42 (4.88)	59 (3.59)	
	4	6 (0.69)	12 (0.73)	
	5	2 (0.23)	2 (0.12)	
	6	1 (0.11)	0	
Mean Stent Diameter (mm)		2.84 ± 0.43	3.01 ± 0.46	0.0001
Patient with Small vessel Disease (</= 2.5 mm)		539 (62.7)	714 (43.51)*	<0.0001
Patient with long lesion		519 (60.4)	960 (58.5)	0.35
Long Lesion		485 (56.46)	911 (55.5)	0.65
Patients with LL in Saphenous Vein graft (SVG)		335 (38.99)	429 (26.1)*	<0.0001
Lesion LL in SVG (mm)		305 (35.5)	389 (23.7)*	<0.0001

Abbreviations: PCI, coronary artery bypass; LVEF, Left ventricular ejection fraction; CHF, congestive heart failure; IHD, ischemic heart disease; MI, myocardial infarction; ACS,acute coronary syndrome ; STEMI, ST elevation myocardial infarction; NSTEMI, non-ST elelvation myocardial infarction

The 3P-MACE rate at 1 and 2 years were captured and are tabulated as Table 2 and Table 3 respectively. Overall, 7 patients were lost during 24 months’ follow-up of which 5 of them were diabetics and the remaining 2 were non-diabetics. The MACE rate at 1 year and 2 years in the overall population was 2.9% and 3.16% respectively. It was mainly driven by TLR/TVR rate of 1.36% (1 year) and 1.44% (2 year). It was found that at both at 1 and 2 years, the 3P-MACE was higher (*P* < 0.05) in diabetic patients as compared to non-diabetics driven primarily by higher CV deaths. Although patients with diabetes showed slightly higher rate of ST but the difference was not statistically significant.

**Table 2:** Outcome indicators at1 year follow up

**Cumulative Results at 1 Year follow-up**	**Overall (2493) N (%)**	**DM (854) N (%)**	**Non-DM (1639) N (%)**	***P*** ** value**
Lost to follow up	7 (0.28)	5 (0.58)	2 (0.12)	0.038
**3-P MACE**	73 (2.9)	36 (4.2)	37 (2.3)	0.009
Cardiac-Death	30 (1.2)	16 (1.9)	14 (0.9)	0.044
TV-MI	9 (0.36)	4 (0.5)	5 (0.3)	0.774
TLR/TVR	34 (1.36)	16 (1.9)	18 (1.1)	0.165
**Total Stent Thrombosis**	14 (0.56)	7 (0.8)	7 (0.4)	0.34
Definite	10 (0.4)	5 (0.6)	5 (0.3)	0.477
Acute stent thrombosis	2 (0.08)	1 (0.1)	1 (0.1)	0.78
Sub-acute stent thrombosis	8 (0.32)	4 (0.5)	4 (0.2)	0.575
Late stent thrombosis	0 (0)	0 (0)	0 (0)	-
Probable	4 (0.16)	2 (0.2)	2 (0.1)	0.894
Acute stent thrombosis	4 (0.16)	2 (0.2)	2 (0.1)	0.894
Sub-acute stent thrombosis	0 (0)	0 (0)	0 (0)	-

Abbreviation: TLR/TVR, target lesion/vessel revascularization; DM, diabetes mellitus; MACE, major adverse cardiovascular events; ST, stent thrombosis; TV-MI, target vessel myocardial infarction *P* values refer to the two subgroups.

**Table 3:** Outcome indicators at 2 year follow up

**Cumulative Results at 2 Year follow-up**	**Overall (2493) N (%)**	**DM (854) N (%)**	**Non-DM (1639) N (%)**	***P*** ** value**
Lost to follow up	7 (0.28)	5 (0.58)	2 (0.12)	0.038
**3-P MACE**	79 (3.16)	40 (4.7)	39 (2.4)	0.002
Cardiac-Death	34 (1.36)	18 (2.1)	16 (1)	0.034
TV-MI	9 (0.36)	4 (0.5)	5 (0.3)	0.774
TLR/TVR	36 (1.44)	18 (2.1)	18 (1.1)	0.069
**Total Stent Thrombosis**	14 (0.56)	7 (0.8)	7 (0.4)	0.34
Definite	10 (0.4)	5 (0.6)	5 (0.3)	0.477
Acute stent thrombosis	2 (0.08)	1 (0.1)	1 (0.1)	0.78
Sub-acute stent thrombosis	8 (0.32)	4 (0.5)	4 (0.2)	0.575
Late stent thrombosis	0 (0)	0 (0)	0 (0)	-
Probable	4 (0.16)	2 (0.2)	2 (0.1)	0.894
Acute stent thrombosis	4 (0.16)	2 (0.2)	2 (0.1)	0.894
Sub-acute stent thrombosis	0 (0)	0 (0)	0 (0)	-

Abbreviations: TLR/TVR, target lesion/vessel revascularization; DM, diabetes mellitus; MACE, major adverse cardiovascular events; ST, stent thrombosis; TV-MI, target vessel myocardial infarction *P*values refer to the two subgroups.

## Discussion

The findings of the present study assessing performance of Abluminus DES+ can be broadly summarized as:

1. In a large “Real world experience” population with minimal exclusion criteria, the efficacy/safety profile of the Abluminus DES+ is confirmed with very low rates of adverse events both at immediate and intermediate-term follow up till 24 months.
2. Though the 3P-MACE in diabetic subgroup was relatively higher than non-diabetics, it was primarily driven by higher CV mortality with comparable event rates in terms of TV-MI and ST, makes it an effective alternative to currently available DES.

With India being touted as “Diabetic capital” of world and estimated disease burden of 69.9 million by 2020, it is expected to impart mammoth of clinical and economic task on healthcare system. Diabetes is a complex chronic disease with a challenging subset of population developing myriad cardiac events. Various recent publications have shown that more than 25% of patients referred for PCI or coronary artery bypass graft (CABG) procedures are diabetics.^7-11^ Though it is reported that PCI and CABG improve survival, reduce risk of MI, stroke and also improves quality of life in diabetic patients, this improvement is often claimed to be inferior as compared to their non-diabetic counterparts especially with respect to in-stent restenosis, Stent thrombosis, myocardial infarction (MI) and all-cause mortality as well as cardiovascular death.^12-14^ Patients with DM have nearly 3 times higher MACE in the early period (0–1 year) but these findings are not observed in the late period (1–2 year).^15^

The Intracoronary Stenting and Angiographic Results: Do Diabetic Patients Derive Similar Benefit from Paclitaxel-Eluting and Sirolimus-Eluting Stents (ISAR-DIABETES) study, of 250 diabetic patients that compared sirolimus-eluting stent (SES) with paclitaxel-eluting stent (PES), had shown that SES arm had lesser late lumen loss and restenosis compared with PES (6.9% vs 16.5%, *P* = 0.03) with repeat revascularization reduced by almost 50% with the use of SES (6.4% vs 12% PES, *P*= 0.12).^16^ These findings were further corroborated by the Intracoronary Drug-Eluting Stenting to Abrogate Restenosis in Small Arteries (ISAR-SMART)-3 study where the late lumen loss with SES was 0.25 mm vs 0.56 mm with PES (*P* < 0.001). These and similar other studies paved the way for most of the newer generation DES using “Limus” platform rather than the “Taxols”.

The intermediate and long-term clinical outcomes of DES and bare-metal stents in diabetic population have already been investigated through the best of the study designs – randomized control trials and prospective registries.^17-19^ One of the other important 300-patient randomized ESSENCE-DIABETES trial succeeded in showing non-inferiority of Everolimus-eluting stents (EESs) compared to first-generation sirolimus-eluting stents with respect to angiographic late lumen loss (LLL) at 8 months with no significant difference in clinical outcomes at 1 year, although the trial was not powered to show a statistical difference with respect to the latter follow ups.^20^ Though the study was a prospective trial assessing MACE at the interval of 12 months only, the event rate was little higher (5.3% with SES) than current study in the diabetic patients (4.2%). Moreover, the follow up duration was also shorter than the present registry. Death rate at 12 months follow up with EES was 1.3% which was comparable with our findings of 1.9%. However, with SES arm in the study, the rate was noticeably higher at 3.3% as compared to our registry. However later, EVOLVE II trial that included a Diabetes sub study (N=203) showed that the 5-year TLF rate was 14.3% for SYNERGY and 14.2% for PROMUS Element Plus (*P* = 0.91) with similar rates at 1-year (*P* = 0.90) and from 1 to 5 years (*P* = 0.94) with no significant differences in the rates of cardiac death, myocardial infarction, or revascularization. Among patients with diabetes mellitus, the target lesion failure rate to 1-year was noninferior to a prespecified goal at 5 years of 17.0%. Thus SYNERGY was comparable to PROMUS Element Plus, with low rates of stent thrombosis and MACE at 5 years of follow-up.^21^ A pooled analysis of 6,780 patients treated with second-generation EES versus first generation paclitaxel-eluting stents enrolled in the SPIRIT II, SPIRIT III and SPIRIT IV and COMPARE randomized trials showed that, despite improved safety and efficacy of EES in non-diabetic patients at 2 years, there was no difference between the devices with respect to outcomes in diabetic patients (n=1,869).^22^ The mortality rate at 2 years irrespective of the stent was similar to the current registry (2.1%), however other indicators of MACE are considerably lower in the current study (TV-MI – 0.5 vs 4.4%; TVR/TLR – 1.44 vs 8.9/5.4%). Furthermore, different second-generation DES devices – utilizing permanent or bioresorbable polymers – have not demonstrated differential efficacy in patients with diabetes.^23,24^

**Figure 1:**
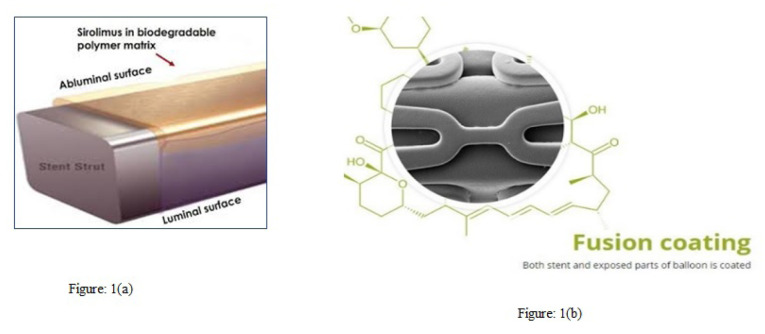
Dual coating of the stent a) Abluminal coating b) Fusion coating

The Abluminus DES+ (Envision Scientific, Surat, India) has an abluminal coating of PLLA/PLGA (50:50 Lactide-co-glycolide) polymer matrix on the stent abluminal surface and exposed balloon surface in pre-crimped configuration (Figure 1). The pre-crimped stent is spray-coated with solution of Sirolimus and polymers mixture in appropriate solvent. Combination of these features with a required prolonged inflation up to 30 seconds during the implantation, ensures a biphasic drug release with an initial burst of 40-50% of the drug release during the first 3-4 days, and then a controlled release up to 48 days.^25^ The exposed parts of the balloon actually work as a drug coated balloon (DCB), releasing sirolimus at the time of implantation. At present, sirolimus and its derivatives are largely applied to stent platforms only unlike Abluminus-DES + thus making it unique platform from other DES.

In the diffuse diabetic disease pattern, drug diffusion plays an important role. Uniform distribution of drug from the stent and the balloon makes the anti-proliferative drug available to the deficient part of stent and which is hypothesized to lower the restenosis rate in diabetic patients. Evidence suggests that Sirolimus inhibits Nuclear Factor-kappaB (NF-kB) – a key inflammatory substance that is released upon device induced injury by the balloon and the stent deployment.^26^ This kinetics of initial burst of drug release followed by a sustained controlled release of sirolimus could actually explain the consistent results observed in the present registry across the different subgroups of patient with or without diabetes as compared to the other similar generation DES.

Abluminal biodegradable polymer SES was evaluated in Cordimax platform and was noninferior to Cypher select for in-stent LLL, in-stent mean diameter stenosis at 9 months’ angiographic follow-up with MACE rates not different at 1 year (5.9% vs 4.0%, *P* = 0.376); but lower MACE rates from 2 to 5 years in the Cordimax group (6.8% vs 13.1%; *P* = 0.039) thus highlighting that MACE rates from 2 to 5 years were less in the abluminal biodegradable polymer group.^27^ A randomized controlled trial showed MACE of 3.3% with EES and 10.0% with ZES in diabetes during 6 months follow up which was far greater than the MACE observed in present registry.^28^ A meta-analysis of 13 randomized trials conducted by Mahmud et al (2008) showed that substantially higher risk of cumulative as well as individual event rates in diabetes when compared with our findings.^29^ 334 Indian Diabetic patients implanted with BioMatrix stent followed up to 24 months had a MACE rate of 1.27% with definite stent thrombosis only in 2 patients.^30^ Based on this hypothesis, a large international registry on diabetic patients is actively enrolling (DEDICATE trial) targeting 5000 patients in Europe and Asia. Yet another randomised controlled trial comparing the angiographic and clinical performance of Abluminus DES+ versus Everolimus eluting stents at 6 months (ABILITY trial) is on-going. The critical findings obtained from this 2-year registry may provide key insights into the efficacy and safety of Abluminus-DES+ in both diabetic and non-diabetic patients and effectively guide future randomized controlled trials. By far the results with the Abluminus-DES+ are promising with considerably low overall 3P-MACE and stent thrombosis rates at 1 and 2 years. The active follow-up of nearly all the enrolled patients and reasonable sample size makes these findings robust and reliable. The higher 3P-MACE rate in diabetics is explicitly driven in the study by higher CV mortality. The higher CV mortality in diabetics can partly be explained by higher prevalence of traditional CV risk factors. Apart from higher age in diabetic patients (59.4 ± 10.85 vs 56.9 ± 10.85; *P* < 0.001), diabetic cohort had higher prevalence of hypertension (59.6 vs 33.15%), dyslipidemia (8.73 vs 4.26%) and pre-existing renal diseases (3.37 vs 1.84%). Lower baseline LVEF in diabetics as compared to non-diabetics (39.12 ± 12.22% vs 47.52 ± 8.86%; *P* = 0.0001) and past history of previous cardiac revascularization (PCI, CABG) are also likely responsible for higher CV deaths in diabetic patients as compare to non-diabetics. This was also compounded by smaller stent diameter and longer stents implantation (*P* < 0.0001) in diabetes. However, despite smaller vessel diameter and longer stents, TV-MI and TLR were not higher in diabetics. This observation prompts for the need of future studies focusing primarily on the diabetics with Abluminus DES+ stents.

## Conclusion

The current prospective registry of Abluminus-DES+ establishes it as safe and effective DES with considerably good outcomes in the diabetics as well as non-diabetics with low 3P MACE rates in the cohort. 3P-MACE driven by CV events was higher amongst diabetics but TV-MI and TLR were comparable with non-diabetics making it a feasible choice amongst diabetics undergoing PCI.

## Acknowledgements

Authors acknowledge the contribution of all the participating Investigators and their teams of each center for their contribution in the study. We also acknowledge the contribution of Envision scientific and its team in the manuscript.

## Competing interest

AllAuthors except Dr. Kamal Sharma were part of the en-ABL-e registry and their sites received funding as per the approved agreements.

## Ethical approval

This was Post-marketing surveillance study of Abluminus stent which is approved by DCGI for usage for coronary angioplasty. Theinstitutional Ethics committee approvals were obtained prior to initiation of the study. The study has been performed in accordance with the ethical standards as laid down in the 1964 Declaration of Helsinki and its later amendments or comparable ethical standards. This article does not contain any studies with animals performed by any of the authors.

## Funding

The study was funded by Envision Scientific, Surat, India.
